# Functional Charge Transfer Plasmon Metadevices

**DOI:** 10.34133/2020/9468692

**Published:** 2020-01-30

**Authors:** Burak Gerislioglu, Arash Ahmadivand

**Affiliations:** ^1^Department of Physics & Astronomy, Rice University, 6100 Main St, Houston, Texas 77005, USA; ^2^Department of Electrical & Computer Engineering, Rice University, 6100 Main St, Houston, Texas 77005, USA

## Abstract

Reducing the capacitive opening between subwavelength metallic objects down to atomic scales or bridging the gap by a conductive path reveals new plasmonic spectral features, known as charge transfer plasmon (CTP). We review the origin, properties, and trending applications of this modes and show how they can be well-understood by classical electrodynamics and quantum mechanics principles. Particularly important is the excitation mechanisms and practical approaches of such a unique resonance in tailoring high-response and efficient extreme-subwavelength hybrid nanophotonic devices. While the quantum tunneling-induced CTP mode possesses the ability to turn on and off the charge transition by varying the intensity of an external light source, the excited CTP in conductively bridged plasmonic systems suffers from the lack of tunability. To address this, the integration of bulk plasmonic nanostructures with optothermally and optoelectronically controllable components has been introduced as promising techniques for developing multifunctional and high-performance CTP-resonant tools. Ultimate tunable plasmonic devices such as metamodulators and metafilters are thus in prospect.

## 1. Introduction

Plasmonics is a promising counterpart of nanophotonics which has witnessed major progresses in the control and manipulation of electromagnetic (EM) fields at extreme-subwavelength scales [1–3]. Central to all studies is the surface plasmon's unique capability to harvest, process, and concentrate light and convert it into energetic near-fields, thermal power, and hot carriers [4–7]. All these remarkable applications and developments of subsequent optical devices have been realized using resonant plasmonic nanostructures across the ultraviolet (UV) to the far-infrared spectra. Resonances in nanophotonics and nanoplasmonics are the fundamental phenomena that play a critical role in defining the operating mechanism, quality, and performance of the tailored tools. In recent years, several types of radiating and nonradiating resonances and spectral effects have been successfully excited and introduced by scientists such as Fano resonances [8–10], electromagnetically induced transparency (EIT) [11–13], Bormann and Kerker effects [14–20], toroidal multipoles [21–24], anapoles [23, 25–27], and charge transfer plasmons (CTPs) [28–30]. Excluding the later instance (CTP), other resonances can be induced based on robust coupling of optically driven modes between proximal metallic nanoparticles (NPs) with sharp protrusions in the near-field regime. Conversely, for the CTP, the charge transfer in particle plasmon resonances has been reported in (1) conductively linked NPs [31–35], (2) reversible electrochemical compounds [36], and (3) metallic systems with subnanometer atomic openings (Fowler-Nordheim (FN) and quantum tunneling principles) [37–40].

There have been ongoing theoretical and experimental advancements to understand the possibility of direct dynamic charge transport between plasmonic objects in the absence of capacitive coupling between plasmonic particles. Relatively, researchers have shown the possibility of plasmon-driven charge transfer process in DNA-mediated metallic NP dimers [31, 34]. In this context, one can fill the gap between metallic NPs with DNA (as a scaffold for the growth of the NPs within the gap), and by increasing the metallic NP concentration, the transition from capacitive to conductive coupling can be formed. Besides, for the conductively bridged NPs, it is confirmed that due to the shuttling of photoexcited electrons across the junction, the CTP spectral feature appears in the lower energies far from the superradiant dipole moment [41]. On the other hand, as an alternative route to this method, it is well-accepted that particles with atomic gaps in between (below ~0.5 nm) are able to sustain CTPs through molecular quantum tunneling principle [40, 42]. Theoretically, the electron tunneling across an atomic-scale gap at optical frequencies cannot be explained using classical electrodynamic theory and requires quantum mechanical description.

In spite of possessing interesting properties, CTPs are inherently suffering from lack of spectral tunability. Recent demonstrations that the functionality of CTP feature can be optimized by integrating bulk metallic systems with thermally and electronically controllable materials show why tunable CTP spectral features are important for implementing novel and advanced plasmonic tools [36, 43–46]. More precisely, this shortcoming has successfully been addressed by combining plasmonic structures with, for example, phase-change materials (PCMs) [43, 44, 47], graphene [45, 46], and thermally tunable substances (i.e., InSb).

In this review, we summarize recently introduced mechanisms that underpin the electron transition between plasmonic particles and trending applications of functional CTPs. One of the goals of this article is to present a comprehensive depiction of various modalities developed for the excitation of tunable CTPs. Besides, we explain the state-of-the-art approaches that have been proposed for enhancing the tunability of CTPs and the use of this spectral phenomenon in designing practical photonic tools such as modulators, switches, and nonlinear harmonic signal generators. Next, we illustrate how a careful integration of subwavelength plasmonic resonators with optically, thermally, and electronically functional materials leads to the emergence of multifunctional nanooptoelectronic and terahertz (THz) devices.

## 2. Charge Transfer Plasmons in Conductively Bridged Particles and Resonators

Direct manipulation of charges *via* redistribution and transfer has been introduced as a simple approach to excite CTP spectral features and studied both theoretically and experimentally in nanoscale plasmonic systems [31–36]. Possessing a different principle compared to capacitive resonance interference, the direct charge transfer between plasmonic resonators and particles enables tailoring advanced plasmonic tools. Additionally, this direct transition of charges between the metallic structures delivers exceptional abilities to actively control the charge shuttling by varying the intensity of the incoming beam, which could be used for the development of near-infrared and THz plasmonic devices.

**Figure 1:**
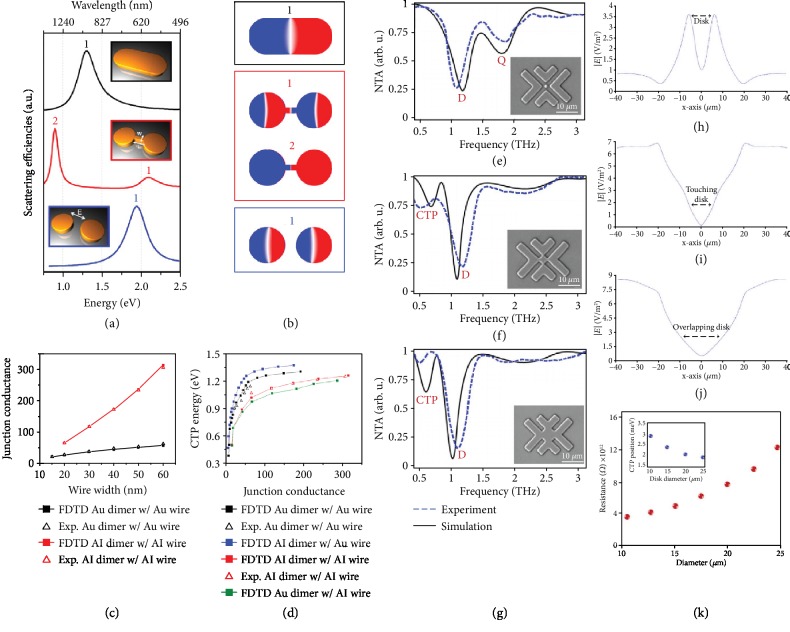
(a) Numerically computed scattering efficiencies of a single dimer (blue), a conductive nanowire-linked dimer (red), and a nanorod (black). The insets show the schematics of the studied nanostructures. The diameter of the disk is 95 nm, the width and the length of junction wire in the junction are 15 and 30 nm, and the thickness of all structures is 35 nm. (b) Charge plots at the position of scattering peaks for the structures in (a): a dipolar plasmon for the nanorod, capacitively coupled superradiant dipolar resonance (1) and CTP resonance (2) for the nanowire-bridged dimer, and a CTP resonance for the dimer. (c) Experimentally and numerically obtained junction conductances of nanowire-bridged dimers at CTP resonances as a function of nanowire width for aluminum and gold nanostructures. (d) CTP resonance as a function of the junction conductance for nanowire-bridged dimers with varying junction substances [32]. Copyright 2016, American Chemical Society. Characterized and simulated normalized transmission spectra for the metallic assembly for the presence of (e) a nanodisk between gaps, (f) a touching disk to the V-shaped resonators, and (g) an overlapping disk. Insets are the corresponding SEM graphs for disk diameter variations. (h–j) Cross-sectional *E*-field concentration (∣*E*∣) diagrams for the presence of a nontouching disk, presence of a touching disk, and presence of an overlapping disk in the middle of the unit cell, respectively. (k) Junction resistance variations as a function of the intermediate disk diameter. Inset is the CTP position as a function of conductive disk diameter [33]. Copyright 2016, Optical Society of America.

**Figure 2:**
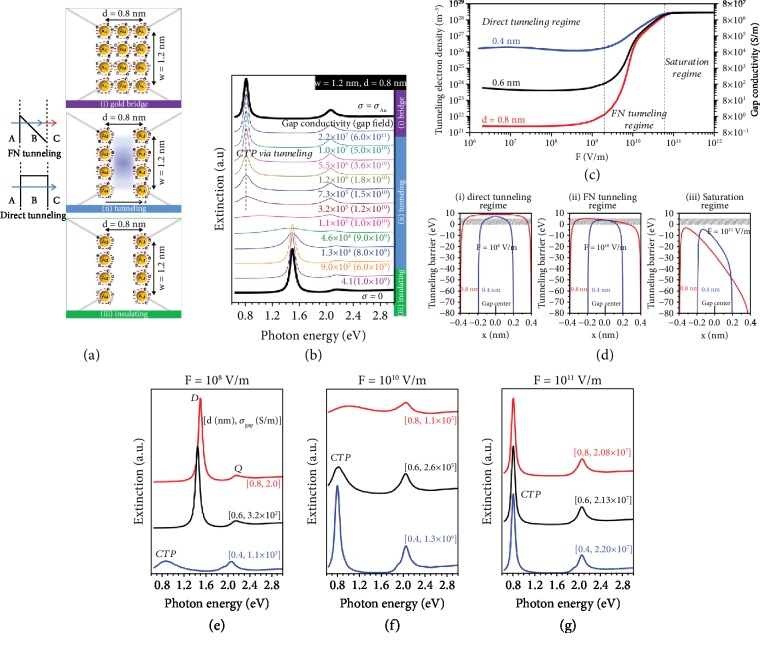
(a) Schematics of considered regions as conductive gold (the charge transfer track consists of free electrons and gold atoms), conductive gap (the charge transfer path is dominated by either direct or FN tunneling electrons), and insulating gap (the charge transfer route is empty). (b) The simulated extinction spectra of these three regions for a possible range of gap conductivities. (c) The calculated gap conductivity and tunneling electron density as a function of the intensity of the applied electric field for three different gap sizes: 0.8 nm, 0.6 nm, and 0.4 nm. (d) The tunneling barrier profiles within the gap for three possible regimes: (i) direct tunneling, (ii) FN tunneling, and (iii) saturation. In all regimes, the shaded areas indicate the energy levels of the tunneling electrons. (e–g) The simulated extinction spectra for diverse compositions of gap lengths and fields. For the weak field condition in (e), 0.6 nm and 0.8 nm gap sizes could not support the CTP mode, but they can underpin hybridized dipolar (*D*) and quadrupolar (*Q*) modes [39]. Copyright 2012, American Chemical Society.

A remarkable example for the excitation of CTP mode is observed in a two-member dimer consisting of a pair of proximal metallic NPs that are connected with a conductive junction (insets in Figure 1(a)) [32]. In the method developed by Wen et al. [32], a dimer antenna with a conductive junction has been considered as the intermediate case between a conventional metallic dimer with capacitive opening and a nanorod. This allows to understand the formation of new spectral features due to the presence of a metallic bridge between NPs. Figure 1(a) demonstrates the numerically obtained scattering efficiencies of three different structures with judiciously defined geometries that are stated in the figure caption. Under *p*-polarized beam illumination, in the dimer limit, the capacitive coupling between the two nanodisks leads to strong hybridization of plasmons and excitation of a superradiant mode around ∼1.95 eV. On the other hand, bridging the dimer with a conductive nanowire gives rise to the formation of two peaks in the spectrum correlating with the dipolar mode (at ∼2.1 eV) and a narrow CTP mode at lower energies far from the classical dipole (around ∼0.96 eV). Lastly, for the plasmonic nanorod, compared to the dimer structure, the dipolar resonance is red-shifted to ∼1.3 eV. As can be seen in the charge plots in Figure 1(b), the fundamental difference between the CTP extreme and dipolar mode is the distinguished oscillation of the electric current density in the junction of the conductive nanowire-bridged plasmonic dimer, while there is no such a feature in both dimer and nanorod structures. Moreover, the charge distribution at the energy of CTP explicitly represents the splitting of charges across the structure (see Figure 1(b), (2)).

To further study the role of the conductive junction on the excitation of CTP, Wen and teammates have shown that the junction geometry and conductance strictly determine the position and intensity of the CTP feature. To this end, the researchers employed CTP-resonant nanostructures based on aluminum and gold substances. Considering a nanowire with the frequency-dependent AC conductivity of *σ*(*ω*), and specific length (*l*), width (*w*), and thickness (*t*), the corresponding conductance can be written as

(1) $$\begin{array}{c} G=\sigma \left(\omega \right)\frac{wt}{l}. \end{array}$$

As a critical parameter, in Figure 1(c), the junction conductance as a function of nanowire width is plotted, numerically and experimentally. Obviously, by increasing the width of the junction, the relative conductance increases slightly for gold structure, while this value sharply increases for the aluminum nanowire. Here, for structures with the equivalent geometries of the bridging nanowire, aluminum structures hold a higher junction conductance than gold structures. Further analysis for the junction properties and plotting the CTP position as a function of junction conductance reveal that the CTP mode exhibits a dramatic sensitivity to the junction conductance in the small conductance limit (Figure 1(d)). However, this sensitivity becomes strikingly less as the conductance increases beyond a specific value (~50) for all the studied cases.

Recent studies have also shown that CTP mode can be viewed as a spectral feature at much lower energies (e.g., far infrared, THz) when the coupling between neighbor resonators is in the weak regime. As a leading work in the THz regime, Ahmadivand et al. [33] have represented the transition from capacitive coupling to direct charge transfer using a symmetric cluster of V-shaped metallic microblocks. The insets in Figures 1(e)–1(g) illustrate the scanning electron microscopy (SEM) images of the unit cell, where the diameter of the central disk gradually increases. As obvious in the normalized transmission amplitude (NTA) profile (Figure 1(e)), the capacitive coupling plays a fundamental role in the emergence of two pronounced minima in the spectral response. The deeper minimum around 1.22 THz correlates with the dipolar resonant mode, while the quadrupole mode appears as a weaker dip around 1.85 THz. Here, the middle disk enhances the strength of the induced multipolar modes via the dipole-dipole interaction [48]. To continue, increasing the diameter of the disk monotonically and providing a touching regime between the disk and V-shaped assemblies lead to the elimination of the quadrupolar moment and emerging of the CTP dip around ~0.5 THz (Figure 1(f)). Due to the formation of insignificant touching spots between the blocks and the central disk, one can expect the direct flow of charges across the unit cell similar to the optical systems and quantum transitions. Further increases in the diameter of the central disk to realize the overlapping regime give rise to a damping in the energy of both dipolar and CTP modes projected from the spectral characteristics of the entire unit cell (Figure 1(g)). The cross-sectional electric-field (E-field) intensity along the unit cell axis helps to perceive the role of the conductive disk in the excitation of CTP feature. As plotted in Figures 1(h)–1(j), by inserting and increasing the size of the disk, the E-field confinement reduces dramatically owing to losing the capacitive coupling between the V-shaped blocks. Additionally, the overlapping areas between the V-shape and central resonators allow to induce sharper and boosted plasmonic dipolar and CTP modes in the THz limit. The geometry of the merging regions enables the shuttling of charges between the linked V-shaped pixels of a standalone assembly. This can be better understood by defining the resistance (*R*) of the disk as a function of the junction geometry [49]:

(2) $$\begin{array}{c} R=\frac{2}{\pi t\sigma \left(\omega \right)}\ln\left(\frac{2d}{\delta }\right), \end{array}$$

where *σ*(*ω*) is the frequency-dependent AC conductivity, *d* is the length between junctions, and *δ* is the contact width at the junctions due to overlapping. Moreover, according to Ohm's law, the conductance is defined by [50]

(3) $$\begin{array}{c} \sigma =\frac{n_{m}e^{2}\tau }{m_{e}}, \end{array}$$

where *n*_*m*_ is the electron density of metal, *e* is the elementary charge, *τ* is the mean-time between collisions, and *m*_*e*_ is the mass of electron. Enlarging the diameter of the overlapping disk lengthens the distance between junctions, and the corresponding resistance increases accordingly (Figure 1(m)). Although the dynamic charges would be able to transit across the trail, the dissipative losses can be significant because of the inherent disk resistance. When the incident THz beam is resonant with the spectral line shape, the fairly strong confinement of charges across the lossy metallic resonators leads to a drastic electron decay. Thus, by extending the size of the middle disk, the induced dipolar and CTP modes shift towards the lower energies due to the enhanced dissipative absorption losses [51, 52].

## 3. Charge Transfer Plasmons in Nonlocal and Quantum Regimes

As discussed in Introduction, the general idea to model the light-matter interactions and the resulting electromagnetic field distribution is mainly based on solving coherent charge density oscillations using classical electromagnetic theory by applying Maxwell's equations [4]. By shrinking the interparticle distance down to nanoscales, localization and intensity of the incident electric field can be enhanced [53–55]. Nevertheless, when the system requires to operate beyond the nanometer gap regime (i.e., subnanometer openings) or contains a quantum topology (e.g., quantum dots, molecules, or atoms), nonlocal screening effects (due to quantum nature of electron) modify the plasmonic response of structure [56]. In this limit, quantum mechanical effects (e.g., collective quantum electron tunneling, nonlocal transport) occur and the whole system must be described under nonlocal conditions [57–59], by implementing advanced theoretical approaches [60–62]. This enables the calculation of the confinement of the induced surface charges appropriately [63]. Such computations in quantum systems have successfully been done using a quantum-corrected model (QCM) [42]. Basically, this concept is an updated version of the time-dependent density-functional theory (TDDFT), which combines the classical electromagnetic framework with the collective quantum electron tunneling [38]. As declared in Savage et al. [64], when the interparticle distance is *d* ≥ 0.4 nm, one can treat the system using classical Maxwell's descriptions to understand the plasmonic interactions. For *d* < 0.4 nm, classical formulation starts to diverge as a result of the increased rate of critical electron tunneling between proximally located NP surfaces (~ 0.3 nm), where the plasmon interactions start to get into the quantum regime. For *d* < 0.3 nm, the quantum tunneling dominates the spectral response of the entire system. By considering a rectangular barrier, this phenomenon can be predicted as the following: ${\tilde{\;d}}_{QR}=\ln\left(3q\alpha \lambda /2\pi \right)/2q$, where ${\tilde{d}}_{QR}$ is the critical gap size, *q* is the semiclassical electron tunneling wavenumber, *λ* is the optical plasmon wavelength, and *α* is the structural constant. More realistically, the addition of “coherent quantum transport” parameter into the formula given above boosts the tunneling rate, which gives rise to the quantum-tunneling CTPs (more detailed information about the calculations can be found in Ref. [64]). In the upcoming part, we briefly review the recent advances in the quantum-mechanical charge shuttling process in nearly touching metallic NPs.

As a pioneer work, Wu et al. [39] demonstrated the first theoretical explanation of plasmon resonances between extremely close NPs due to FN tunneling. In spite of possessing certain conditions to satisfy the direct tunneling (e.g., subnanometer gap), this approach provides a novel outlook to induce CTP modes for a different set of gap sizes by applying high-intensity illumination. From the active nanophotonic device perspective, the proposed concept is significant owing to its ability to control the charge shuttling process by changing the intensity of the incident light. As discussed previously, approaching two NPs to each other (nearly touching regime) and the presence of a metallic path between them lead to transition of oscillating electric charges across the nanoplatforms, in addition to the dipolar mode of the dimer system. As the conductive path becomes narrower, the excited CTP resonance shifts to the longer wavelength and several higher-order modes (e.g., hybridized dipolar and quadrupolar modes) appear [65–67]. Nevertheless, when the conductive bridge turns out to a ~0.4-0.5 nm gap, the CTP mode disappears, meaning that a quantum mechanical framework should be taking into account to model possible electron tunneling effects. To this end, in Figure 2(a), the authors demonstrated possible tunneling mechanisms as direct tunneling (effective for small interparticle distance) and FN tunneling (dominant when a strong electric field is applied) in a simple schematic. For the direct tunneling, electrons tunnel through the square barrier (from A to C); while for the FN tunneling, electrons first tunnel through the triangular barrier (from A to B), then transport to C. In general, the electrons exist in B are called “space charges.” Hence, this process can also be termed as space charge-based charge transfer. Moreover, the researchers here utilized the microscopic version of Ohm's law (see Equation (2)) to specify the tunneling electrons in the gap [68]. It is important to note that for the calculation of tunneling electron density in the gap, Wu and colleagues applied the Kohn-Sham density functional theory, to model exchange correlation and Coulomb interactions between electrons [69, 70]. Further, they successfully explained the resulting CTP mode in light of the obtained tunneling electron density (*n*_gap_) and gap conductivity (*σ*_gap_), which are determined based on a numerical methodology developed by the authors [39]. To briefly discuss, *ε*(*ω*) = 1 + *i*(*σ*_gap_/*ωε*_0_) is utilized as a part of QCM to derive the optical response of the system. As demonstrated in Figure 2(b), when *σ*_gap_ is larger than 1.145 × 10^5^ S/m, a CTP mode is emerged. For the “tunneling” part indicated in Figure 2(b), a large plasmon-enhanced electric field (10^10^ V/m) (or “gap field”) is required to bring enough electron in the space charge area to induce the CTP resonant mode. In the following, in Figure 2(c), to better understand the physical mechanism behind the process, *σ*_gap_ and *n*_gap_ are compared for the gap sizes of 0.4 nm, 0.6 nm, and 0.8 nm in three different gap field regimes. In the direct tunneling limit, the tunneling barrier can be controlled through the charge potential when the gap field is small, which means *σ*_gap_  primarily rely on the gap length. As expected, when the gap is 0.4 nm, electrons can straightly tunnel along the narrower barrier (see Figure 2(d), (i)), with the increased tunneling probability of electrons, but for larger gaps (e.g., 0.8 nm), the charge flow process could not be sustained because of the wider tunneling barrier. To address this issue, the employed electric field intensity can be amplified (~10^10^ V/m) to start the FN tunneling procedure, in which the tunneling barrier is reshaped as plotted in Figure 2(d), (ii). In this regime, *σ*_gap_ and *n*_gap_ are very responsive to the intensity of the external illumination, meaning that these parameters can be easily modified depending on the applied electric field intensity. For instance, when the field intensity is altered from 10^9^ V/m to 10^11^ V/m, *σ*_gap_ is increased from 1 to 10^7^ S/m. Any further increase in the field intensity would lower the level of barrier where all tunneling electrons can stay over the barrier (see Figure 2(d), (iii)). In this regime, *σ*_gap_ is not dependent on the applied field intensity and gap size, as illustrated in Figure 2(c). This results in perfect transmission of electrons through the gap and extremely large *σ*_gap_ to retain the CTP mode for the studied gap dimensions. Lastly, in Figures 2(e)–2(g), simulated extinction spectra for different gap size (*d*), *σ*_gap_, and the gap field are presented to prove the tunability of the CTP peak. With the gap size of 0.4 nm, the CTP mode always arise either *via* direct tunneling (Figure 2(g)) or *via* FN tunneling (Figure 2(f)). For larger gap sizes (e.g., 0.6 nm and 0.8 nm), high intensity external irradiation (~10^10^ or 10^11^ V/m) is necessary to excite the CTP resonant mode (see Figures 2(f) and 2(g)).

**Figure 3:**
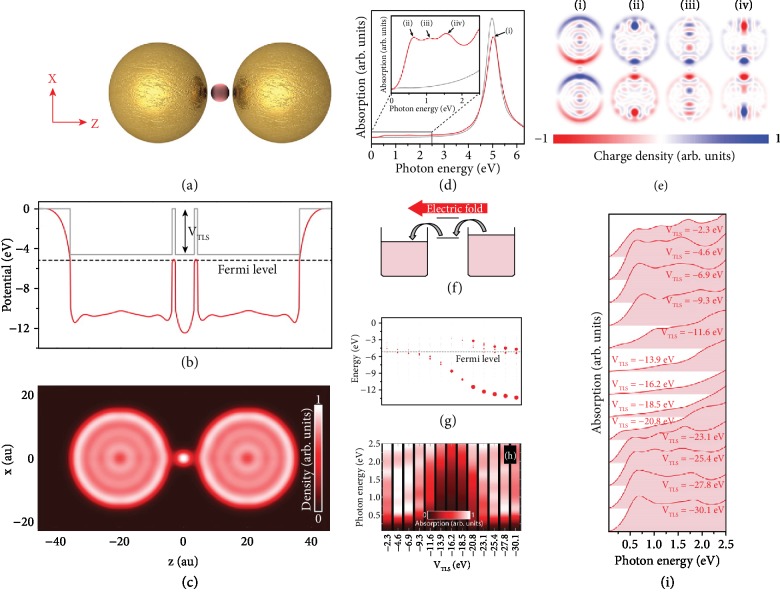
(a) An artistic sketch of the studied two-level system. (b) One-electron potential (red) and background potentials in equilibrium. The corresponding Fermi level of the nanoplatform is represented by a dashed black line. (c) Equilibrium electronic density of the given system. (d) The calculated absorption spectrum for both bare (gray) and TLS added (red) dimer. Inset: zoomed-in version of the low-energy area. (e) Corresponding charge plots of the induced modes at (i) 5.05 eV, (ii) 0.65 eV, (iii) 1.10 eV, and (iv) 1.55 eV. (f) A schematic to denote the physical mechanism behind the generation of CTP mode. (g) The electronic structure of the system as a function of background potential of TLS. The black dashed line displays the Fermi level of the platform. (h, i) Two different versions of the absorption spectra of the system as a function of *V*_TLS_ [71]. Copyright 2015, American Chemical Society.

In another recent study, Kulkarni and Manjavacas [71] investigated the quantum effects related to the charge transfer process by examining the spectral response of gold dimer with a two-level system (TLS) (e.g., an atom or a molecule). In this understanding, fully quantum-based computations showed that CTPs are only recognizable if one of the energy levels of the studied two-level system is in resonance with the Fermi level of the dimer system, by enabling the electron transition along the junction. At resonance, the absorption spectrum of the system and the conductance of the junction are calculated, and the outcomes indicated that the conductance of the junction is equivalent to one quantum of conductance, which is *G*_0_ = 2*e*^2^/*h*. In Figure 3(a), a schematic for the studied subwavelength system is illustrated. This system consists of two identical gold spheres of 32 au in diameter and a small sphere with a diameter of 6 au to model the TLS. In all calculations, the whole platform is assumed to be in a vacuum. To explore the capabilities of the system, TDDFT in the adiabatic local density approximation [72, 73] is utilized. To this end, the authors only take into account the conduction electrons of the metal and the gold NPs are modelled based on jellium approximation, where the ionic background charge has a uniform charge density (*n*_0_). Here, *n*_0_ is judiciously selected to correlate with the density of gold, which is equal to a Wigner-Seitz radius of 3 au. In a similar fashion, TLS is formed by having a Wigner-Seitz radius to make sure that TLS only adds a single electron to the system. Without applying any external field, the one-electron potential of the platform is defined as *V*_eff_(**r**) = *V*_0_(**r**) + *V*_H_[*n*(**r**) − *n*_0_(**r**)] + *V*_xc_[*n*(**r**)], where *V*_H_ is the Hartree potential, *n* is the electronic density, *V*_xc_ is the exchange-correlation potential (also known as the Perdew-Zunger functional as a part of local density approximation [74]), *n*_0_ is the background ionic charge density, and *V*_0_ is the uniform background potential which incarcerate the electrons within the NPs and the TLs. For the gold NPs, this potential is fixed at -4.6 eV (5.2 eV lower than the vacuum level) to be able to have a proper electron spill-out. As plotted in Figure 3(b), the energy levels of the TLS are tuned according to the Fermi level of the NPs. Additionally, the background and the resulting equilibrium one-electron potentials are represented as gray and red colors, respectively, for *V*_TLS_ = −4.6 eV. It is evident from Figure 3(b) that the potential barrier of the junction (indicated as dashed black line) is reduced by virtue of the TLS. In the following, the associated equilibrium electronic density is demonstrated in Figure 3(c), which clearly indicates Friedel oscillations due to the discretization of the electronic levels [74]. Besides, the electronic density in the junction is a proof of the single energy level of the TLS. Although the TLS decreases the potential barrier, the electronic density in the gaps between the NPs does not reach to zero. In Figure 3(d), the authors plotted the absorption spectrum for two different cases (by considering, the incident field is polarized along the *z*-axis): (a) the bare metallic dimer (gray line) and (b) the dimer with the TLS (red line). For the latter case, the background potential of the TLS is selected as equal to the NPs. In both cases, a bonding dipolar mode (BDP) [75, 76] appears around 5 eV. For the low-energy part of the spectrum, three new peaks are formed, due to the addition of TLS into the dimer system. To comprehensively evaluate the origin of these modes, the corresponding charge distributions are studied on the surface of the NPs (see Figure 3(e)). The results clearly show that the mode around 5 eV has a dipolar nature, and the other three modes indicate a monopolar distribution pattern, which is the solid evidence of the charge transfer through the junction. What is more, the modes at 1.1 eV and 1.55 eV display an oscillation of charges inside the NPs owing to the finite size effects. Based on these results, one can state that the only way to induce a CTP in this system is contingent upon the use of the levels of the TLS as the conductive paths (see the schematic in Figure 3(f)). Thus, the authors analyze the effect of background potential of the TLS on the response of the system. As demonstrated in Figure 3(g), *V*_TLS_ is varied to manipulate the position of the levels of the TLS. In this plot, each dot represents an energy level of the whole platform. Depending on the localization rate of the TLS levels, the size of the dot can be bigger, which is beneficial to differentiate localized levels in the TLS. A group of states are localized close to the Fermi level of the system, shown in black dashed line, for small numbers of *V*_TLS_. These states become more localized as the background potential is getting deeper and the states move to lower parts in the energy diagram. However, because of the strong localization, the interplay between the NPs is diminished. Particularly, when  *V*_TLS_ is lower than -16.2 eV, an extra level starts to localize on the TLS. Lastly, in Figures 3(h) and 3(i), the absorption spectrum of the system (at a low-energy part) is presented for the *V*_TLS_ values considered in Figure 3(g). The results explicitly verify the requirement of having a localized state in the TLS, whose energy is near the Fermi level of the system, to be able to generate a CTP mode.

## 4. Functional Charge Transfer Plasmons

As discussed in the previous section, in the quantum tunneling of energetic optically driven electrons, possessing an active control over the CTP spectral feature is limited to modifying the incident field intensity and/or morphological variations [42, 45]. On the other hand, the induced CTPs *via* the direct transition of optically driven electrons across the bulk metallic paths between NPs suffer from the inherent lack of tunability. Recently, these challenges have effectively been addressed by using optoelectronically and optothermally tunable components in the purpose of the excitation of functional CTPs [43–47, 77]. Such an active tunability allows for the exploration of several integrated plasmonic instruments and applications owing to its great potential for the next-generation multifunctional technology. Beyond the fundamental studies, now research in CTP devices has been focused on the experimental attempts to efficiently transform its capabilities into the real-world applications, where two fundamental issues must be addressed: functionality and scalable fabrication. While the former has been successfully realized by employing optoelectronically and thermally tunable compounds, in considering the later concern for industrial applications, one of the major challenges is scalable cost-effective fabrication of the functional metastructures. This can be done, for instance, by developing robust techniques based on nanolithography-free techniques. In this section and following subsections, we briefly review the recent techniques that have been utilized to optimize the tunability of CTPs. We will demonstrate how the integration of the plasmonic nanostructures with optoelectronically and optothermally controllable components improves the tunability of CTP spectral features.

### 4.1. Graphene-Enhanced Functional CTP Devices

A one-atom thick layer of *sp*^2^-hybridized carbon atoms, known as graphene, has received copious interest due to its significantly high electron mobility, mechanical flexibility, and exquisite optical properties [78–82]. Graphene-enhanced fundamental applications include but not limited to the light harvesting [82–84], ultrafast optics [85–87], nonlinear photonics [88–90], and quantum effects [91, 92]. One of the most interesting properties of 2D carbon sheet is the possibility of controlling the photoconductivity of this monolayer *via* modifying the generated carrier density [93, 94]. This exquisite advantage has effectively been accomplished by modeling the electronic properties of graphene in terms of massless Dirac fermions [93–95]. This feature enables graphene to display strong infrared plasmons and made it as a promising component in implementing advanced nanophotonic devices [96–100]. The tunable AC photoconductivity of graphene allows to provide semimetallic behavior with an optical conductivity as a function of quantum conductance as [93, 94]

(4) $$\begin{array}{c} \sigma =\frac{\pi e^{2}}{2h}, \end{array}$$

where *h* is Planck's constant. Analogous to the plasmonic components, the spectral properties and plasmonic response of graphene sheet can be estimated by the Drude absorption model for a wide range of carrier densities [93, 94, 101]. To describe the free carrier photoconductivity with parabolic dispersion in a 2D sheet, one can demonstrate the temperature-independent model as [68, 102]

(5) $$\begin{array}{c} \sigma \left(\omega \right)=\frac{{ne}^{2}}{m\left(\Gamma -i\omega \right)}, \end{array}$$

in which *m* is the electron mass and Γ is the transport scattering rate. The unprecedented levels of beam confinement and EM field enhancement by graphene allow for having electrostatic control over the plasmonic response in the absorption spectra [96]. The successful example of such quantum effect was provided to explain the quantum effects in the plasmonic response of graphene nanostructures linked by a thin molecular junction. Thongrattanasiri and colleagues [77] demonstrated that the intrinsic characteristics of graphene enable to tune the absorption spectra of a dimer structure *via* adding small number of atoms. Using first-principle analyses, the plasmonic response of the entirely graphene-based dimer was studied for the intermediate junction with varying atomic row widths (4-8). The designed triangular structures forming the bowties are oriented with respect to the graphene lattice by assuming having armchair edges. This prevents the possible losses that can be naturally observed in zigzag-edge nanostructures, due to the presence of zero-energy electronic edge states [103].

**Figure 4:**
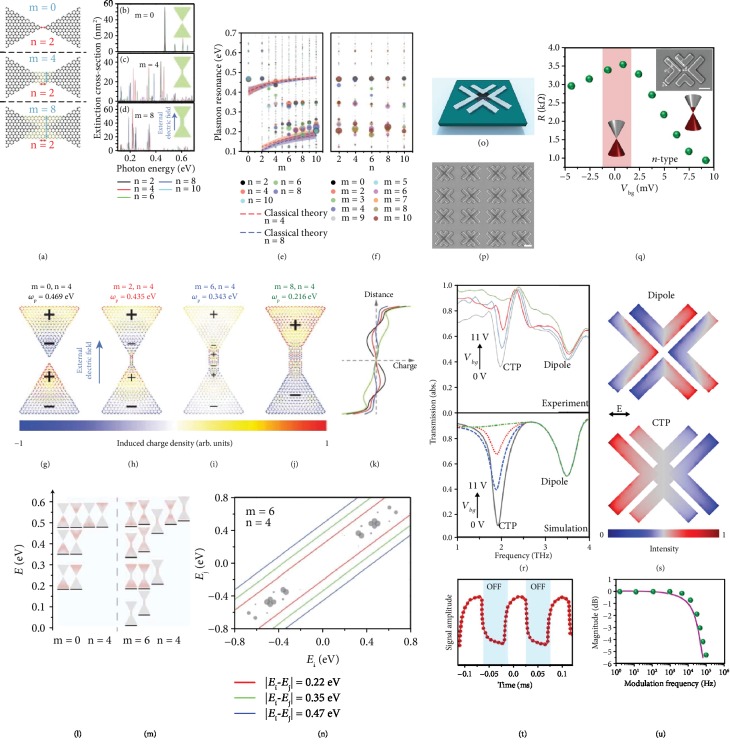
(a) Details of the junction region in the graphene structures, with definitions of junction length *n* and width *m*. (b–d) Computed spectra for particular bridge widths *m* = 0, 4, and 8 and several other lengths as indicated by different colors. The insets show the complete graphene structures for *n* = 2. (e) Exhibited plasmon resonances from the graphene structure as a function of bridge width *m*. The color code for different lengths *n* is given in the upper inset. The area of the circles is proportional to the area under the extinction peak for each plasmon feature. Conventional plasmon energies are indicated using dashed curves for *n* = 4 (red) and *n* = 8 (blue), bordered by shaded areas, representing the strength of the modes. (f) Plasmon resonances as a function of junction length *n*. The graphene is doped to a Fermi energy *E*_*F*_ = 0.4 eV and has a mobility *μ* = 10,000 cm^2^/V·s, and the length of the bowties is 8 nm. (g, h, i, j) Charge density maps with the color of each atom indicating its induced charge for different junction widths *m* = 0, 2, 6, and 8 and the same length *n* = 4. (k) Induced charge integrated along the horizontal direction and averaged over four nearest carbon-atom neighbors. (l, m) Electron density distribution of electronic states in *n* = 4 bowtie structures for *m* = 0 ((l), separated triangles) and *m* = 6 ((m), linked triangles). The energies of these states are shown by black lines under the density plots. (n) Dipole matrix elements between electronic states of the same bowtie as in (m). The area of the circles is proportional to the dipole strength [77]. Copyright 2013, WILEY-VCH. (o) Schematic of the graphene island-mediated THz cluster. (p) SEM image of the fabricated device. The scale bar is 10 *μ*m. (q) Resistance variations of the graphene monolayer, obtained numerically from the source-drain current with *V*_SD_ = 25 mV. The inset is the magnified SEM image for the fabricated sample to introduce the geometrical parts as follows: *a*/*b*/*c*/*d* = 3.5/10/7.5/4 *μ*m. The thickness of the metallic blocks is set to 200 nm, and the scale bar is 5 *μ*m. (r) Normalized transmission amplitude of the graphene-plasmonic structure under applied gate voltage. (s) The current density maps at the dipole and CTP resonance frequencies for the graphene monolayer in dielectric (*V*_bg_ = 11 V) and conductive (*V*_bg_ = 0 V) regimes. (t) The responding optical signal of the tunable device under fast on/off THz radiation modulation. (u) Experimentally (circles) and numerically (solid line) determined normalized modulation magnitude (dB) [46]. Copyright 2019, Royal Society of Chemistry.

To begin with, Thongrattanasiri et al. [77] used the random-phase approximation (RPA) theory and finite-element method (FEM) to analyze the plasmonic response of the graphene-based bowties. In addition, by fixing the fix side length of the triangles to 8 nm in all cases (almost ∼10^3^ atoms in each triangle), the researchers utilized the following settings: Fermi energy of the structures was to *E*_*F*_ = 0.4 eV, the intrinsic damping as *ħτ*^−1^ = 1.6 meV, all corresponding to a DC mobility of 10^4^ cm^2^/V·s [104, 105].  Figure 4(a) demonstrates the details of the bowties and junction area for three different characteristic values of the bridge width based on the number of carbon-atom zigzag rows (*m* = 0, 4, 8). It should be underlined that when *m* = 0, this means there is no atomically connection between proximal nanotriangles. Figures 4(b)–4(d) represent the spectral response of the structures for varying junction length (*n*). As shown in Figure 4(a), *n* resembles the number of carbon hexagons that are required to join the graphene triangles for *m* = 2. As can be explicitly seen in the extinction cross-section, by increasing the junction width, the spectral features show a trend from higher to lower energies.  Figure 4(e) shows this effect in details, in which the plasmonic features are arranged as a function of energy and junction width for different values of the junction length *n*. The size of the circles is prepared proportional to the intensity of the plasmon mode defined as the area under the corresponding plasmon peaks in the extinction profile. In the narrower junction limit, the spectra point out pronounced plasmonic features placed around ∼0.47 eV. On the other hand, when the junction becomes wider, the spectra are dramatically dominated by lower-energy plasmonic features around ∼0.22 eV. Finally, for the junction width intermediate geometries, there is a complex transition between the mentioned two regimes, with intermediate-energy features around ∼0.35 eV. As depicted in Figure 4(f), the length (*n*) of the junction does not have a significant influence on the spectral changes. It is significant to note that the three distinguished behaviors of the plasmon energies declared before for all narrow, intermediate, and wide bridges perform for all sizes of the junction length.

Further analyses help to understand the nature and properties of the plasmons in the graphene-based structures. In Figures 4(g)–4(l), the induced charge distribution profiles are plotted from narrow to wide junctions, while the length (*n*) of the junction was fixed. Relatively, Figure 4(g) illustrates the polarization profile for the high energy plasmons (∼0.47 eV) in the capacitive coupling regime (nontouching condition, *m* = 0), validating a dipole-dipole interference that is not altered when a narrow junction (*m* = 2) is inserted between the two triangular resonators of the structure (see Figure 4(h)). Conversely, when the width of the molecular bridge increases towards the intermediate regime (*m* = 6, with the energy of plasmons around ~0.35 eV), one can see a distinct dipolar polarization pattern across the junction (see Figure 4(i)). In this limit, the junction plasmons become the dominant feature, arising from the local electronic properties of the junction. Lastly, for the wider bridge (*m* = 8), similar to the fully metallic structures studied previously, the CTP becomes the dominant spectral response (see Figure 4(j)). The signs on the density plots of charge distribution maps qualitatively correspond to the distribution of the plasmon-induced charge as a function distance to the dimer center, which is exhibited in Figure 4(k). Interestingly, increasing the length (*n*) of the junction between neighbor graphene-based nanotriangles leads to effects that are consistent with the conclusions for the width of the bridge. Strictly speaking, Thongrattanasiri and teammates verified that the classical description of the investigated graphene nanostructures fails to regenerate the plasmonic behavior derived from first principles. This is obvious, for instance, for the junction width with intermediate sizes, the conventional computations oversight the intermediate-energy junction plasmons that have been observed in the quantum calculations. In addition, another disagreement between classical and quantum modes has been observed, in which the traditional approach predicted a smooth fadeout of the dipole-dipole mode exhibited by nonoverlapping triangles during insertion of the bridge, while the CTP shows a singular behavior, as it migrates towards zero energy in the limit of vanishing junction width.

In Figure 4(l), it is illustrated that the electronic states contributed in the plasmon of separated graphene nanotriangles are almost equal to the plasmons from an individual nanopixels. However, this involves a minor amount of Coulombic interaction and hybridization of plasmons. By insertion of a junction with an intermediate width, the strength of the hybridized modes increases significantly, leading to the emergence of novel electronic junction states (see Figure 4(m)). The noteworthy point here is the observation of two new junction plasmons around the zero energy (Dirac point). This was anticipated by researchers, because of the presence of carbon zigzag edges in the molecular bridge [106–108]. Therefore, in the junction plasmon regime, the excitation of plasmons contains electron or holes in the corresponding electronic junction states.  Figure 4(n) demonstrates the strength of these electron-hole pair dipole transitions in the graphene-mediated structure as a function of initial and final energies (the area of circles demonstrates the strength of the electron-hole pairs). As can be explicitly seen in this panel, the plasmon energies (shown by solid curves) do not overlap with the dominant electron-hole transports. This energy mismatch reveals that the optical transitions are not single-particle excitations. This strongly supports the claim of collective plasmonic nature of the bowtie optical excitations. The advantage of this technique is the possibility of tuning the doping level of molecular graphene nanobridge, which enables possessing an active control over the charge transition. This results in the excitation of tunable spectral features such as functional CTPs.

To continue, we briefly consider the recent advances in enhancing the functionality of CTP resonances based on graphene-mediated metallic metastructures. Newly, Ahmadivand and colleagues have developed an approach to induce CTPs in gate-controlled graphene monolayer-integrated particle clusters in the THz spectra towards tailoring multifunctional metamodulators (see the schematic in Figure 4(o)) [46]. Using the exquisite AC photoconductivity of graphene island [94], the researchers demonstrated that the dynamic frequency-dependent conductivity of graphene has a direct relation to the extinction in the transmitted wave, which is defined by [109, 110] 1 − *T*/*T*_0_ = 1 − 1/|1 + *Z*_0_*σ*_AC_(*ω*)/(1 + *n*_*s*_)|^2^, where *Z*_0_ is the vacuum impedance, *n*_s_ is the refractive index of the thin substrate, and the optical conductivity of graphene (*σ*_AC_(*ω*)) can be taken by using the RPA principle. This leads to the strong capacitive coupling of plasmons at the *charge neutrality point* of graphene islands (resembling high resistance regime). This results in the excitation of a typical electric dipole. On the other hand, when the back-gate voltage is applied, the low resistance of graphene islands gives rise to the direct charge transition between the metallic resonators via the conductive atomic bridge and the excitation of THz-CTPs.  Figure 4(p) exhibits the SEM image of the fabricated assemblies in periodic arrays with the presence of aligned graphene islands at the middle spot between the aluminum V-shaped blocks, which was implemented on a multilayer SiO_2_/ITO substrate. Here, the ITO sublayer acts as a conductive surface for the applied voltages *via* the gate to control the AC photoconductivity of graphene islands. The resistance variations for graphene area in the presence of metallic objects are measured as a function of back-gate bias (*V*_bg_), shown in Figure 4(q). The charge neutrality point of graphene islands was determined by neglecting the inherent contact resistance of the electrodes (*V*_bg_^cnp^ = 9.5 V). Moreover, the maximum resistance was measured around ~3.52 k*Ω* corresponding to the dielectric regime, while the lowest resistance was monitored for the graphene islands in the *n*-type phase correlating with the conductive regime. The geometrical parameters of the proposed unit cell are superimposed in the inset of this panel.

To demonstrate the spectral properties of the device, firstly, the researchers computed and measured the spectral response under longitudinal polarized THz beam exposure at room temperature (300 K), as depicted in Figure 4(r). In these sets of analyses, the researchers judiciously changed the applied bias to the gate in order to control the transition of charges across the graphene-mediated unit cell. As plotted in both panels, a distinct dipolar mode is excited around ~3.5 THz due to the capacitive coupling between the neighboring metallic resonators. The dipolar mode in all different regimes is unchanged because of its intrinsic independency from the charge transfer between the blocks. Additionally, for graphene islands in the *n*-type doping limit, the atomically thin junction between metallic V-shape resonators turns to a conductive component and enables the transition of photoinduced electrons across between the blocks. This results in the excitation of a pronounced CTP spectral feature around ~1.95 THz. For high bias regime, the central graphene islands exhibit high-resistance and capacitive coupling becomes dominant, giving rise to the elimination of CTP mode due to blocking of the charge transfer.  Figure 4(s) illustrates the charge distribution map for both dipolar and CTP modes, obtained by the FEM method. Theoretically, such a unique functionality was understood and achieved by implementing the intraband AC conductivity of carbon monolayer [102, 111]. The intraband AC conductivity of graphene is containing both Drude-like and nonzero conductivities at the charge neutrality point. Hence, careful tuning of this parameter would be possible by adjusting the Fermi energy level “*E*_*F*_” based on applying back-gate bias, given by [28] *E*_*F*_ = *ħν*_*F*_ (*πC*_*A*_*V*_bg_), where *ħ* is the reduced Planck's constant, *v*_*F*_ is the Fermi velocity (10^6^ m/s), and *C*_*A*_ is the capacitance per unit area per charge of the multilayer substrate under the graphene islands. This enables active tuning of the frequency-dependent intraband photoconductivity of graphene by modifying the applied back-gate bias. Consequently, one can directly tune the conductance, resistance, and reactance of the graphene-enhanced bridge.

One other area that deserves special attention is that of optical modulation. Optical and optoelectronic metamodulators have previously been designed based on conventionally resonant structures such as Fano and EIT resonant metamolecules [112–117]. The dramatic dissipative and inherent losses correlating with the classical resonant nanostructures have triggered researchers to substitute these metasurfaces with a new type of ones that are capable to provide much faster and efficient modulation properties such as toroidal resonances [24, 115, 118, 119] and CTP spectral features [43–46]. In relation to the latest research by Ahmadivand et al. [46], THz plasmonic metamodulators are strategic optical components that have faced fundamental restrictions such as low efficiency, slow operating speed, and lack of tunability to manipulate THz waves. So far, various methods have been used to address these shortcomings towards designing of high-responsive, efficient, and fast plasmonic modulators. Although some of these techniques were effective, the tailored devices do not provide ultrafast switching and high modulation depth. In the research by Ahmadivand and colleagues, the devised gated graphene-mediated plasmonic device exploits the remarkable electrical and optical features of both graphene and metallic unit cell to enhance the intensity and tunability of the induced resonant spectral feature. In Figure 4(t), the recorded THz signal amplitude from the photodetector under applied bias variations is shown. This was accomplished by sweeping the signal as a function of modulation frequency up to 10^5^ Hz, which led to a remarkable modulation depth up to 72% and fast operation speed with the rising and falling duration around 19 *μ*s and 21 *μ*s, respectively. From the modulation bandwidth principle, Figure 4(u) graph exhibits the normalized modulation magnitude, confirming a 3 dB operation bandwidth of ~19.5 kHz and ~22 kHz for numerical simulations and experimental measurements, respectively.

### 4.2. Phase-Change Material-Enhanced Functional CTP Devices

As elaborated in Sections 2 and 3, inserting a conductive layer underneath a plasmonic nanosystem or reducing the interparticle distance between metallic NPs down to a subnanometer scale leads to the excitation of CTPs at lower energies in the spectrum. Recent efforts have also denoted the use of metallic nanowires between adjacent NPs to make charge flow feasible [32, 120]. For the last two examples, the conductance and the geometry of the conductive junction between plasmonic elements are extremely important to tune the spectral response and the local field distribution of the system. Although the mentioned concepts have provided remarkable outcomes for the future of CTP-based real-world applications, they suffer from the lack of active tunability, which requires solid changes in the optical and electrical characteristics of a given design. One possible way to overcome this issue is the use of chalcogenide phase-change materials (PCMs) as optoelectronically controllable structures. Over the past years, these semiconductor alloys, especially Ge_2_Sb_2_Te_5_ (GST), have attained a copious interest for tailoring plasmonic and all-dielectric platforms from visible to infrared for various purposes, such as switching/modulation [115, 121, 122], sensing [123, 124], and beam steering [125, 126], due to possessing quick phase-changing capability (with a crystallization temperature lower than 477°C), high cyclability, thermal stability, and versatile nonvolatile optoelectronic features between opposite states [127–130]. In the following, we concisely present the use of GST, as a phase-change glass, to develop dynamically tunable and all-optical near-infrared devices to switch between dipolar and CTP resonances based on the phase of GST.

**Figure 5:**
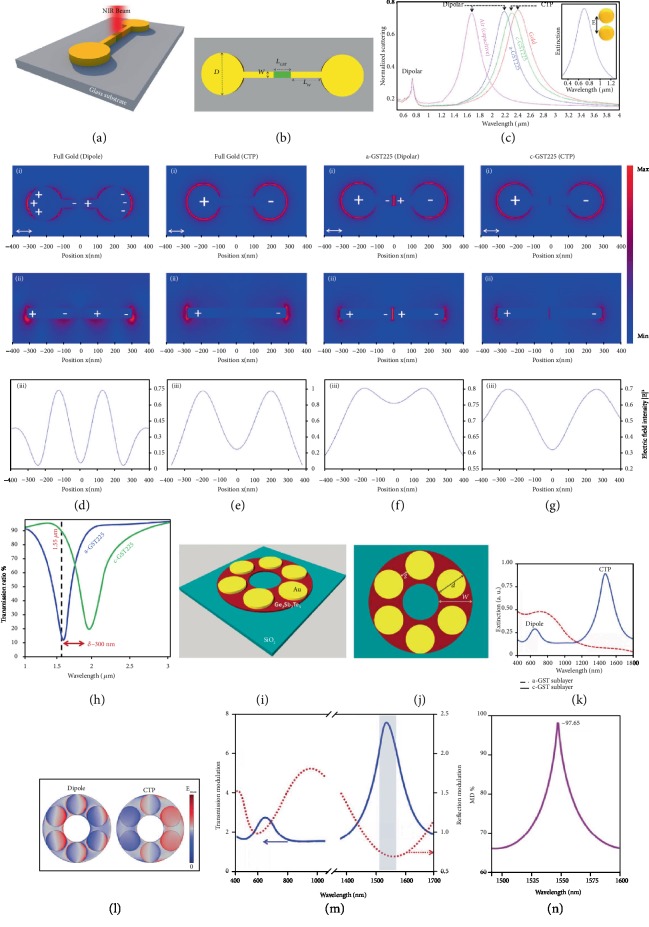
(a) A 3D schematic of the proposed metallodielectric dimer platform. (b) A top-view image of the dimer configuration including the geometrical parameters. (c) Normalized extinction spectra of the bridged dimer for the following conditions: air, a-GST, c-GST, and full gold. (d, e) (i) top-view and (ii) cross-sectional view of the *E*-field maps along the bridged dimer for the dipole and CTP modes in full gold limit, respectively. (f, g) (i) top-view and (ii) cross-sectional view of the *E*-field distributions corresponding to the dipolar (a-GST) and CTP (c-GST) modes. (iii) *E*-field intensity diagrams at the position of each CTP and dipolar modes for all the studied cases. (h) The transmission ratio of the proposed metallodielectric switch in on (c-GST) and off (a-GST) conditions [43]. Copyright 2017, Nature Publishing Group. (i) An artistic sketch and (j) top-view of the studied metallodielectric cluster. (k) Normalized extinction plot of the GST-sublayer mediated hexamer configuration for a-GST and c-GST. (l) Surface charge density plots of the dipolar and CTP resonances for a-GST and c-GST cases, respectively. (m) The transmission and reflection modulation spectra as a function of wavelength. (n) The modulation depth plot of the proposed switch mechanism [44]. Copyright 2017, IEEE.

In Figure 5(a), for the first time, Ahmadivand et al. [43] demonstrated a numerical and theoretical study of a metallodielectric dimer platform as NIR all-optical switch using a PCM. By introducing a GST nanowire into the center of the conductive bridge that links the gold dimer, the researchers obtained remarkable changes in the position and origin of the induced plasmonic modes. To implement this active switching mechanism, Ahmadivand and teammates utilized substantial alterations in both resistivity and permittivity values of GST, owing to optically stimulated phase transition process. The proposed device in this understanding is illustrated in both Figures 5(a) and 5(b), superimposing the geometrical parameters. As mentioned in the earlier parts of this review, the conductivity of the bridging path between NPs is extremely important to generate CTPs. To this end, successful control of the conductivity of the metallodielectric link was achieved by applying the required energy using an additional light source to initiate the phase toggling. Here, the wavelength-dependent conductivity of the GST section was defined as [131] *σ*_GST_(*λ*) = (*c*/2*λ*)(1 − *ε*_eff_(*λ*)), where *ε*_eff_(*λ*) is the effective permittivity of GST portion and *c* is the velocity of light in vacuum. Besides, depending on the crystallization level of the GST, the Lorentz-Lorenz effective-medium description can be performed using [132–134] (*ε*_eff_(*λ*) − 1)/(*ε*_eff_(*λ*) + 2) = *f*_*c*_((*ε*_*c*_(*λ*) − 1)/(*ε*_*c*_(*λ*) + 2)) + *f*_*a*_((*ε*_*a*_(*λ*) − 1)/(*ε*_*a*_(*λ*) + 2)), where *f*_*i*_ is the volume function of the *i*th phase as 0 ≤ *f*_*i*_ = (*n*_*i*_/∑*jn*_*j*_) ≤ 1, where *n*_*j*_ is the density of the *j*th phase. It is worth mentioning that to model the photothermal heating process, we utilized the multicapacitive cascading method [135, 136]. In this approach, the absorbed photothermal heat energy (*E*_H_) in the platform can be defined as [137] *E*_H_ = *AQ*_abs_*F*(*r*), where *Q*_abs_ is the numerical absorption coefficient, *F*(*r*) is the optical fluence of the impinging pulse, and *A* indicates the area of the dimer antenna (more comprehensive information can be obtained from the Supplementary Information file of the article). Furthermore, the normalized extinction spectra of the dimer system are presented for the following four different configurations of the bridge: full gold, air, amorphous GST (a-GST), and crystalline GST (c-GST) (see Figure 5(c)). For completely gold bridged-dimer system, a bright dipolar mode (as a shoulder resonance) is excited around 0.73 *μ*m because of the capacitive coupling between the satellite gold nanodisks. Additionally, the charge shuttling across the conductive junction gives rise to a CTP peak at 2.4 *μ*m. When a small air gap (10 nm in length) is introduced to the monolithic gold nanowire, the charge transfer between the opposite sides of the system is hindered. Similar to the full gold case, a dipolar mode is appeared at higher energies, while a pronounced dipolar extreme is observed around 1.7 *μ*m, owing to the strong capacitive coupling between gold nanorods. Moreover, when the gap area is filled up with GST (initially, considered as a-GST), the dipolar peak around 1.7 *μ*m is red-shifted to 2.2 *μ*m with slightly increased intensity, due to negligible extinction coefficient (*k* ~ 0) of the a-GST in this regime [138]. Once the phase of the GST become fully crystallized (c-GST), its corresponding resistivity value decreases ~6 orders of magnitude and the charge flow starts to dominate the spectral response of the system. As a result, a CTP extreme is formed at 2.3 *μ*m and its position is considerably red-shifted (*δ* ~ 100 nm) in comparison to the dipolar mode of a-GST, because of its absorptive behavior at low energies. It is significant to note that for all the studied conditions, the position and the amplitude of the induced dipolar mode shoulder are not affected by the changes made on the gold nanobridge. To verify this, the extinction profile of a bare gold dimer is plotted in Figure 5(c) as the inset. Next, in Figures 5(d), (i, ii) and 5(e), (i, ii), the differences in the *E*-field distributions are clearly demonstrated the presence of the CTP mode for the full gold structure. In the case of a-GST, opposite charges are accumulated around the dielectric region as well as in the surrounding gold nanodisks (see Figure 5(f), (i, ii)). On the contrary, in the c-GST limit, the intensity of the *E*-field is reduced and the excited charges can still shuttle across the bridge, owing to the minimized capacitive coupling around the GST region in the bridge (see Figure 5(g)). Lastly, the sufficiency of the proposed metallodielectric device for all-optical NIR switching has been analyzed. The corresponding analysis in Figure 5(h) shows that the nanodevice with the length of 100 nm GST portion is the best choice for quick and effective switching purposes with the following features: (a) switching from amorphous to fully crystalline phase in a few ns and toggling back to amorphous case in hundreds of femtoseconds (fs), (b) *δ* ~ 300 nm shift in terms of the resonance point, and (c) 88% of modulation depth at 1.55 *μ*m, which is also known as the telecommunication band.

In another recent study by Nooshnab and Ahmadivand [44], a novel CTP-resonant and optothermally controllable metamodulator is demonstrated. As indicated in Figure 5(i), a six-member gold hexamer nanocluster is placed on top of a ring-shaped GST layer to generate actively tunable plasmonic modes. With this approach, manipulation of the charge transfer process is realized by changing the phase of the GST sublayer. To be able to operate at the telecommunication band, the geometrical parameters given in Figure 5(j) are judiciously selected. In the c-GST limit, a prominent CTP peak is emerged in the vicinity of 1550 nm, owing to the metallic nanocluster and conductive sublayer underneath. Additionally, a dipolar shoulder is formed at higher energies. When the phase of the GST subsurface is reversed to a-GST through optical heating, it acts analogous to a dielectric material and bonding dipolar mode becomes dominant in the spectral response of the system (see Figure 5(k)). In principle, under cylindrical and vortex beam excitations, a plasmonic hexamer can be tailored to sustain pronounced Fano resonances [139, 140]. Nevertheless, in this particular case, the gold hexamer does not support a dark mode due to the plane wave excitation. Furthermore, similar to the previously mentioned work, the authors utilized the Lorentz-Lorenz effective medium theory [132–134] to model *ε*_eff_(*λ*) of the GST sublayer for these calculations. Moreover, optically generated thermal power was quantified using the following equation: *T*(*r*, *t*) = (*AQ*_*a*_*ϕ*/1.77*ντ*)/exp(−((*t* − *t*_0_)^2^/*τ*^2^)) [137], where *τ* is the time constant of the incident light, *ϕ* is the beam fluence, *r* is the distance from the source, and *t*_0_ is the time delay. To verify the origin of the induced modes, the corresponding charge distribution profiles for the proposed device are investigated in Figure 5(l). In the a-GST limit, the excited charges oscillate in the same direction, which is a characteristic signature of the dipolar mode. On the other hand, in the CTP regime, the charges are almost equally separated along the whole structure in connection with the polarization of the impinging light. Next, the modulation performance of the platform is examined. To this end, the reflection and transmission modulation characteristics of the c-GST sublayer based hexamer are plotted in Figure 5(m). Based on the obtained results, one can state that the transition from dielectric phase to conductive phase gives rise to the formation of a new spectral feature around the telecommunication band. Besides, the proposed metallodielectric platform provides a prominent modulation depth (up to ~98%) at the NIR bandwidth (see Figure 5(n)). As a final consideration, the authors pointed out the lossy behavior of the proposed plasmonic system. In light of the calculated insertion loss values, they minimized the possible dissipative losses with the help of the direct charge transfer feature of the c-GST sublayer based hexamer.

## 5. Outlook

In recent years, the field of plasmonics has experienced rapid progresses in understanding the dynamics of conductively bridged and nearly touching metallic NPs. As we described in this focused review, many intriguing physical effects have been predicted in the systems supporting distinguished CTP excitations. So far, various theoretical and experimental investigations have been performed to realize oscillating electric current along the conductive junction, as a key principle of the CTP excitation, depending on the type of the nanosystem. However, related investigations have been limited to the excitation approaches and verifying this principle by standard measurements. Now that the CTP spectral feature has been reached in a broad range of platforms in modern nanophotonics, we believe that it is time to numerically and experimentally explore the modalities towards enhancing the functionality of this spectral phenomenon, eventually, to find its useful and practical applications. We also speculate that CTP states possess a strong potential to make a profound impact in the development of coming generation multifunctional nanophotonic instruments. More precisely, the transition between hybridized dipolar and distinct CTP modes have been explicitly demonstrated under capacitive and conductive coupling regimes, respectively. Highly attractive features of CTP resonances have opened the door for novel active devices and applications, including but not limited to surface-enhanced IR absorption (SEIRA), surface-enhanced Raman scattering (SERS), optical switching, modulation, and waveguiding; however, active manipulation of these resonances using functional materials is overlooked.

## 6. Conclusions

In this review, we briefly presented the recent accomplishments in the use of versatile materials towards actively tunable CTP-based nanoscale tools. As indicated in the considered studies, having an active control on the CTP mode is possible using functional materials like graphene and GST, rather than applying morphological variations. The exceptional optical and electrical characteristics of these materials have allowed researchers to design active CTP-resonant devices. We envisage that this review will provide detailed understanding for the evolution from passive to active CTP-based platforms and pave the road for developing next-generation nanophotonic devices to reach new functionalities.
